# NLRP3 Inflammasome Sequential Changes in *Staphylococcus aureus*-Induced Mouse Model of Acute Rhinosinusitis

**DOI:** 10.3390/ijms150915806

**Published:** 2014-09-09

**Authors:** Yan-Jun Wang, Guo-Qing Gong, Shan Chen, Li-Yan Xiong, Xing-Xing Zhou, Xiang Huang, Wei-Jia Kong

**Affiliations:** 1 Department of Otorhinolaryngology, Union Hospital, Tongji Medical College, Huazhong University of Science and Technology, Wuhan 430022, China; E-Mails: yanjunwangent@163.com (Y.-J.W.); Gong841028@163.com (G.-Q.G.); chenshan1564@126.com (S.C.); xiongliyan0205@126.com (L.-Y.X.); star1028@126.com (X.-X.Z.); 2 Institute of Otorhinolaryngology, Union Hospital, Tongji Medical College, Huazhong University of Science and Technology, Wuhan 430022, China; E-Mail: huangxiang838@163.com

**Keywords:** NLRP3 inflammasome, acute rhinosinusitis, inflammation

## Abstract

The NLR pyrin domain containing 3 (NLRP3) inflammasome plays a crucial role in lung disease and may have a similar role in upper respiratory tract inflammation. We therefore constructed a C57BL/6 mouse model of acute rhinosinusitis induced by *Staphylococcus aureus* and investigated the role of the NLRP3 inflammasome in this model. Mice were classified as non-inoculated group (group A) and inoculated groups (groups B, C, D and E, sacrificed 1, 3, 7 and 14 days after inoculation, respectively). Hematoxylin-eosin staining showed that each group had inflammatory cell infiltration, except group A. The damage of the nasal mucosa was aggravated gradually over time. Western blot and immunofluorescence showed that the structural proteins of the NLRP3 inflammasome (NLRP3, ASC (apoptosis-associated speck-like protein containing CARD), procaspase-1) in groups B, C, D and E were increased gradually. But they were reduced in group B compared with group A, except for NLRP3. Western blot showed that the cleavage fragment of procaspase-1, p20 in groups B, C, D and E was increased gradually. Real-time PCR showed that the corresponding mRNAs of the structural proteins were changed the same as their proteins. IL-1β mRNA and mature IL-1β protein were increased gradually in groups A, B, C, D and E. These results indicate that NLRP3 inflammasome activation was associated with the acute rhinosinusitis, and that there was a positive correlation between the expression level of the NLRP3 inflammasome and the severity of acute rhinosinusitis.

## 1. Introduction

Acute rhinosinusitis is an important health problem that seriously affects the quality of life of sufferers, and represents a considerable economic burden for society [1,2]. Although many studies on the mechanisms of acute rhinosinusitis have been carried out, they are not yet clearly understood.

Recent studies have suggested that the innate immune system may be involved in the development of acute rhinosinusitis [3], due to its mediation of signal transduction through Toll-like receptors (TLRs), which are pattern-recognition receptors (PRRs) [4]. However, recent findings have implied that other PRRs, particularly NOD-like receptors (NLRs), are involved in respiratory tract inflammation [5].

NLRs can associate with other proteins to form protein complexes called inflammasomes [6], and NLR inflammasomes can induce some pro-inflammatory cytokines, such as interleukin (IL)-1β and IL-18 by activating procaspase-1 [7]. The effects of inflammasomes have been studied in other inflammatory diseases, such as crystal arthropathies, periodic fever syndromes and rheumatoid arthritis [8], but the function of NLR inflammasomes in acute rhinosinusitis has not attracted much attention.

The major NLR inflammasomes include the NLRP1, NLRP3 and NLRC4 inflammasomes, and it has been established that the NLRP3 inflammasome is important in the response to various endogenous and exogenous signals [9,10]. The NLRP3 inflammasome consists of NLRP3 protein, the ASC protein and the procaspase-1 protein, and occurs as an NLRP3−ASC−procaspase-1 form. The inflammasome complex is activated by signals that are recognized by macrophages and neutrophils [11,12], after which the procaspase-1 is cleaved into two fragments, *i.e.*, p20 and p10, which activate the pro-inflammatory cytokines (IL-1β and IL-18) to generate the active molecules [7].

However, it has not been clear whether the NLRP3 inflammasome participates in the process of acute rhinosinusitis; we therefore constructed an acute rhinosinusitis model to study this relationship.

## 2. Results and Discussion

### 2.1. Staphylococcus aureus-Induced Acute Rhinosinusitis in C57BL/6 Mice, and the Secretion of Interleukin (IL)-1β

A previous study that attempted to establish a model of acute rhinosinusitis revealed that neutrophil clusters occupied the nasal sinus and that neutrophils infiltrated and damaged the nasal mucosa, as shown by hematoxylin-eosin staining [13]. To test whether the model was successfully constructed, hematoxylin-eosin staining was carried out to determine the histological features of the nasal mucosa and sinus of mice.

A real-time polymerase chain reaction (PCR) was performed to measure the mRNA of the pro-inflammatory cytokine IL-1β, and western blot was used to detect the mature IL-1β protein in the nasal mucosa. Up-regulation of the expression level of IL-1β can be used to provide evidence of inflammation.

**Figure 1:**
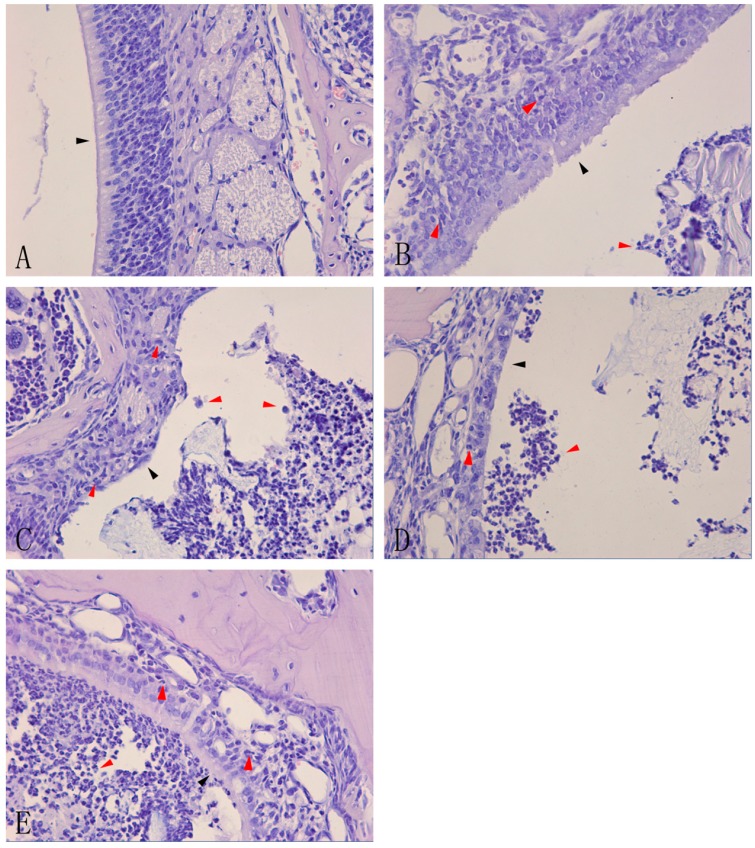
Hematoxylin-eosin staining of nasal mucosa of acute rhinosinusitis induced by *Staphylococcus aureus*.Controlmice remained un-inoculated (**A**). C57BL/6 mice were inoculated in the right nasal cavity with a suspension of *S. aureus* (10μL 1.2×10^9^ CFU/mL) and the inflammation was analyzed after 1 day (**B**); 3days (**C**); 7 days (**D**); and 14 days (**E**).Histological analysis of leukocyte infiltration and morphological analysis of the nasal mucosa in the nasal cavity of mice were carried out by staining with hematoxylin-eosin (×40). The red arrows indicate infiltrated cells, and the black arrows indicate the damage to the nasal mucosa in images (**B**), (**C**), (**D**), and (**E**) and control nasal mucosa in image (**A**).

Histological examination of the nasal mucosa from the control group (group A, *n* = 6) showed normal cells and a normal structure, and no inflammatory cell infiltration (Figure 1A). However, gradually increasing infiltration of inflammatory cells and nasal damage to varying degrees were observed in groups B, C, D, and E (*n* = 6 in each group). A slight infiltration of inflammatory cells and mild damage to the nasal mucosa were observed in group B, with a small number of neutrophils in the nasal sinus and cilia lodging of ciliated cells (Figure 1B). The damage was more severe in group C, with more neutrophils in the nasal sinus, ciliated cell damage, and loss of cilia (Figure 1C). In group D, there was a large accumulation of neutrophils within or around the nasal mucosa, loss of ciliated cells, and thinning of the mucosal layer (Figure 1D). Group E had more neutrophils in the nasal sinus and nasal mucosa than group D, and the ciliated cells were severely destroyed (Figure 1E).

**Figure 2:**
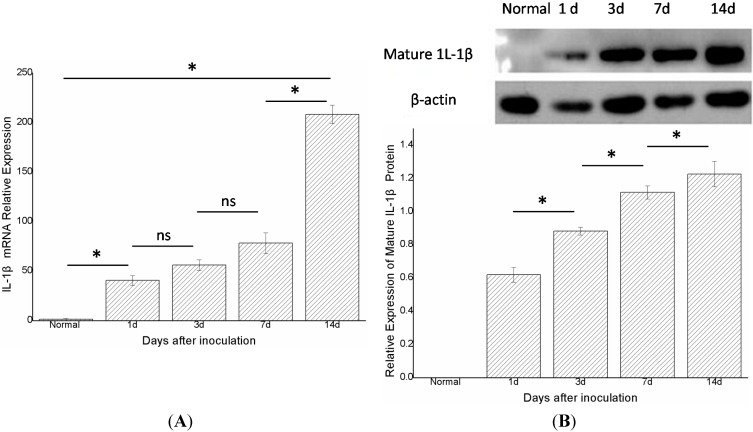
The expression of IL-1βmRNA and mature IL-1β protein in the right nasal mucosa of mice. (**A**) IL-1β mRNA was rarely expressed in the control group; with time, the mRNA levels of IL-1βin the nasal mucosa after 1, 3, 7, and 14 days following stimulationwith *S. aureus* gradually increased;and (**B**) Mature IL-1β protein was not expressed in the control group; after inoculation, the protein levels of mature IL-1βin the nasal mucosa after 1, 3, 7, and 14 days following stimulationwith *S. aureus* gradually increased (*indicates *p* < 0.05; ns= not statistically significant).

Real-time PCR showed that IL-1β mRNA was rarely expressed in group A (*n* = 6). One day after inoculation (group B, *n* = 6), the expression of IL-1β mRNA was markedly increased compared with the control group (group A), and was statistically significant (*p* < 0.05). Furthermore, the expression of IL-1β mRNA in groups B, C, D, and E (*n* = 6 in each group) increased gradually and differed statistically significantly between groups A and B, groups D and E and groups A and E (*p* < 0.05) (Figure 2A). Western blot showed that the mature IL-1β protein was not expressed in the control group, but the level of expression of this protein in the nasal mucosa increased gradually from 1, 3, 7, and 14 days after stimulation, and differed statistically significantly in adjacent groups. (*p* < 0.05) (Figure 2B).

### 2.2 NLR Pyrin Domain Containing 3 (NLRP3) Increased with Time

**Figure 3:**
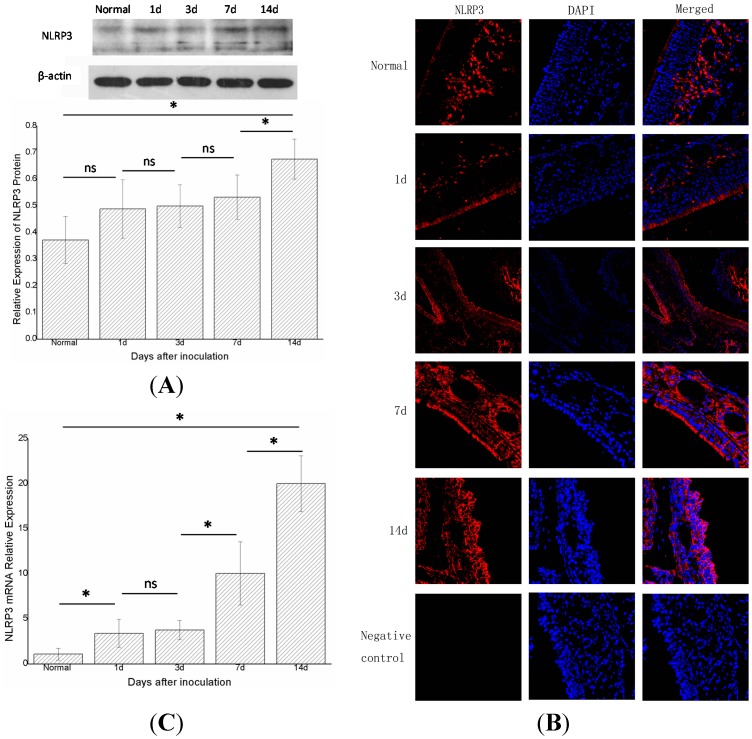
The expression of total NLRP3 protein and NLRP3 mRNA in the right nasal mucosa of mice.(**A**) Western blot assessment of the protein expression of NLRP3 in the control group (group A), and 1, 3, 7, and 14 days (groups B, C, D, and E, respectively) after stimulation with *S. aureus*. A semi-quantitative analysis was used to represent the total protein level of NLRP3; (**B**) Immunofluorescence showed that NLRP3was mainly expressed in the cytoplasm of cells in the nasal mucosa; and (**C**) Real-time PCR assessment of the mRNA expression of NLRP3 in the control group(group A), and 1,3,7, and 14days (groups B, C, D, and E, respectively) after stimulationwith *S. aureus* (* indicates *p* < 0.05; ns= not statistically significant).

The expression of NLRP3 increased to varying degrees with time. At the protein level, western blot showed that the expression of NLRP3 protein in groups A, B, C, D, and E (*n* = 6 in each group) gradually increased and differed statistically significantly (*p* < 0.05) between groups E and D, and groups E and A, (Figure 3A). Immunofluorescence displayed a similar trend (Figure 3B). Real-time PCR showed that expression of NLRP3 mRNA in groups A, B, C, D, and E increased gradually and statistically significantly in adjacent groups, except for groups C and B (*p* < 0.05) (Figure 3C).

### 2.3. Apoptosis-Associated Speck-Like Protein (ASC) Decreased at First and then Increased again

**Figure 4:**
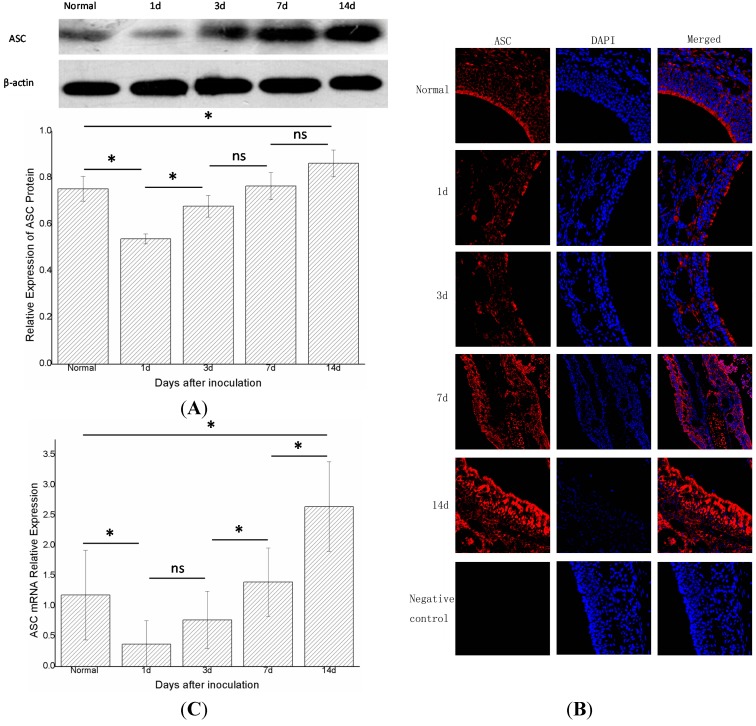
The expression of total ASC protein and ASC mRNA in the right nasal mucosa of mice. (**A**) Western blot assessment of the protein expression of ASC in the control group (group A),and1,3,7,and 14 days (groups B, C, D, and E, respectively) after stimulationwith *S. aureus*. A semi-quantitative analysis was used to represent the total protein level of ASC; (**B**) Immunofluorescence showed that ASC wasmainly expressed in the cytoplasm of cells in the nasal mucosa;and (**C**) Real-time PCR assessment of the mRNA expression of ASC in the control group (group A), and 1,3,7,and 14 days (groups B, C, D, and E, respectively) after stimulationwith *S. aureus* (* indicates *p* < 0.05; ns= not statistically significant).

The changes in ASC did not parallel those of NLRP3. One day after inoculation (group B, *n* = 6), the expression of the ASC protein decreased compared with the control group (group A, *n* = 6) (*p* < 0.05). In groups B, C, D, and E (*n* = 6 in each group), the expression of ASC protein increased gradually with time. Moreover, the levels of ASC protein differed statistically significantly (*p* < 0.05) between groups C and B, groups E and A (Figure 4A). Immunofluorescence showed a similar trend (Figure 4B). Real-time PCR showed that the expression of ASC mRNA in group B declined compared with the control group (group A, *n* = 6) (*p* < 0.05). In groups B, C, D, and E (*n* = 6 in each group), the expression gradually increased with time, and differed statistically significantly in adjacent groups, except for groups B and C (*p* < 0.05) (Figure 4C).

### 2.4. Caspase-1 mRNA Decreased, then Increased; the Protein of the Fragment of Procaspase-1 (p20) Increased over Time

**Figure 5:**
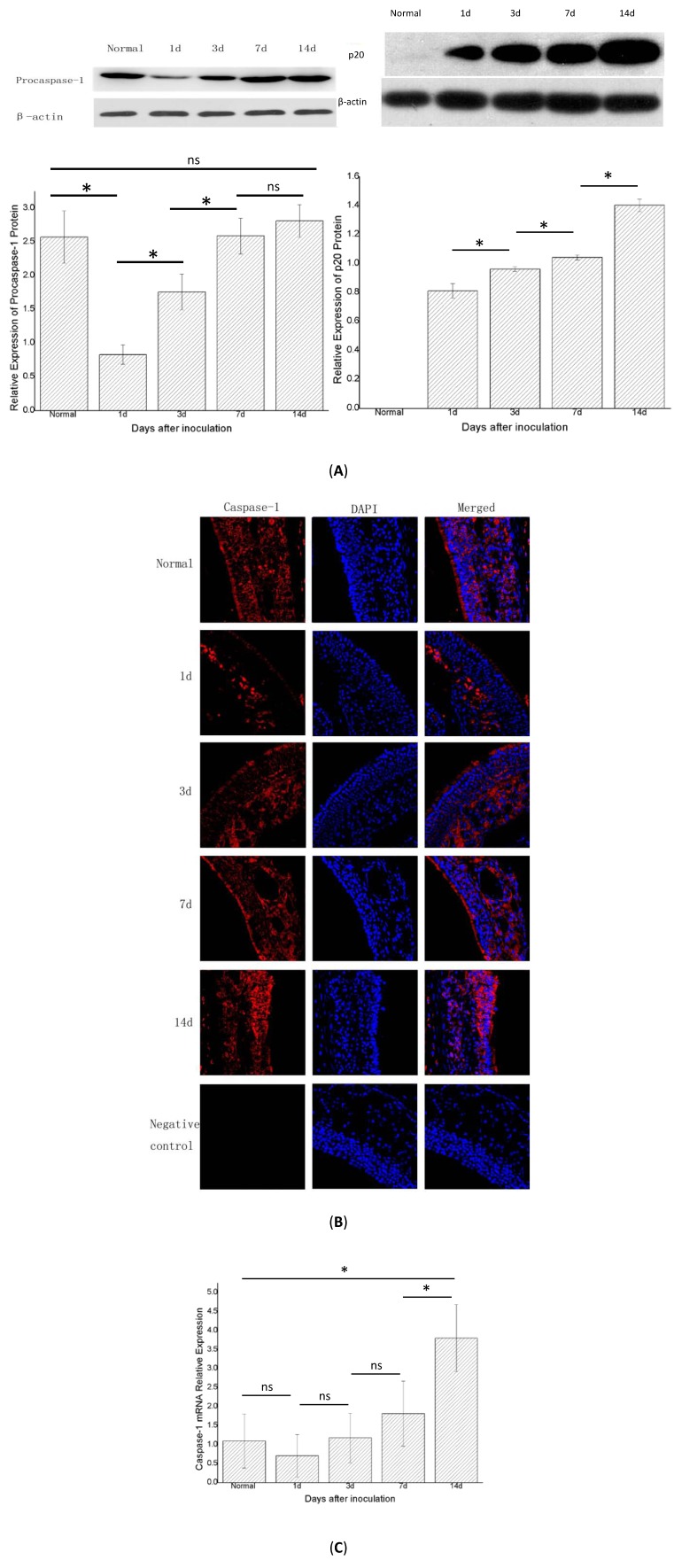
The expression of caspase-1 protein (also called procaspase-1 before caspase-1 was activated) and the fragment of procaspase-1 (p20) and caspase-1 mRNA in the right nasal mucosa of mice. (**A**) Western blot assessment of the protein expression of procaspase-1 and p20 in the control group (group A), and1, 3, 7, and 14 days (groups B,C,D and E, respectively) after stimulation with *Staphylococcus aureus*. A semi-quantitative analysis was used to represent the total protein level ofprocaspase-1 and p20; (**B**) Immunofluorescence showed that caspase-1 was mainly expressed in the cytoplasm of cells in the nasal mucosa; and (**C**) Real-time PCR assessment of the mRNA expression of caspase-1 in the control group (group A), and 1, 3, 7, and 14 days (groups B, C, D, and E, respectively) after stimulationwith *Staphylococcus aureus* (* indicates *p* < 0.05; ns= not statistically significant).

After the NLRP3 inflammasome was activated, procaspase-1 was activated into the form of active caspase-1, which is a tetramer composed of p20 and p10. Therefore, the detection of p20 and p10 can reflect whether the NLRP3 inflammasome has been activated. Western blot showed that procaspase-1 decreased in groups B and C compared with the control group (group A, *n* = 6) and then later increased in group D (*p* < 0.05). In groups B, C, D, and E (*n* = 6 in each group), the expression of procaspase-1 protein was increased and differed significantly in adjacent groups, except for groups E and D (*p* < 0.05) (Figure 5A). Immunofluorescence showed a similar trend (Figure 5B). In this experiment, we measured p20 protein to determine whether the NLRP3 inflammasome was activated. Western blot showed that the p20 protein was not expressed in the control group (group A), but increased gradually in groups B, C, D, and E, and differed statistically significantly between the adjacent groups (*p* < 0.05). Real-time PCR showed that the expression of caspase-1 mRNA in group B was lower than that in the control group (group A, *n* = 6). In groups B, C, D, and E (*n* = 6 in each group), the expression of caspase-1 mRNA gradually increased and differed statistically significantly between groups E and D , and groups E and A. (*p* < 0.05) (Figure 5C).

### 2.5. Discussion

In this study, we constructed a mouse model of acute rhinosinusitis. Hematoxylin-eosin staining revealed ongoing infiltration of inflammatory cells and damage to the nasal mucosa (Figure 1). This result was in accord with previous studies that suggested that the inflammatory cells represented acute rhinosinusitis [13,14]. The gradually increased expression level of IL-1β mRNA and mature IL-1β protein provided more powerful proof that the construction of this mouse model of acute rhinosinusitis was successful.

The mechanisms of acute rhinosinusitis are not clear. In this study, we focused on the innate immune system for it may involve in the process of acute rhinosinusitis, and the innate immune system is generally considered to function via PRRs. To date, the most widely studied PRRs have been the TLRs, such as TLR-2, TLR-4, and TLR-9 [4,15]. However, another type of PRR, called NLR, was related to rhinosinusitis and was found in the cytoplasm. NLRP3 was the most important member of the NLRs [16], and was integrated with ASC through a PYD (pyrin domain)–PYD interaction. The ASC then combined with caspase-1 through a CARD (caspase recruitment domain–CARD interaction. At this time, the caspase-1was not yet activated, and was also known as procaspase-1. The combined NLRP3-ASC-procaspase-1 complex was termed the NLRP3 inflammasome. The combined procaspase-1 was activated to release splitting fragments p20 and p10, which formed a tetramer that was the active caspase-1. The active caspase-1 assisted IL-1β and IL-18 to convert to their mature forms, which were then secreted with the active caspase-1. Thus, p20 expression can reflect whether procaspase-1 is activated. However, in the state of inflammation in mice, not only the NLRP3 inflammasome can active procaspsae-1; there are other pathways, such as the AIM2 (absent in melanoma 2) inflammasome pathway. Therefore, we also investigated NLRP3 and ASC to determine whether the NLRP3 inflammasome participates in the signal transduction. Thus, we measured NLRP3, ASC, procaspase-1 and p20 to determine the expression levels of the assembled NLRP3 inflammasome [17]. Moreover, we investigated the presence of IL-1β mRNA and mature IL-1β protein to verify whether the active caspase-1 is generated. The NLRP3 inflammasome was indicated to play a pro-inflammatory role in this model after inoculation with bacteria; the level of NLRP3 protein increased constantly after inoculation with *S. aureus* (Figure 3A), and the other two components of the NLRP3 inflammasom––ASC protein (Figure 4A) and procaspase-1 protein (Figure 5A) showed the same trend. The results of immunofluorescence were consistent with those of the western blot (Figure 3B, Figure 4B and Figure 5B). These results revealed that the NLRP3 inflammasome was activated after inoculation, and that the expression level of the assembled NLRP3 inflamasome in groups B, C, D and E increased gradually. It could therefore be concluded that the NLRP3 inflammasome was activated in this model, and there was a positive correlation between the expression level of the NLRP3 inflammasome and the severity of acute rhinosinusitis.

Real-time PCR found that the mRNAs of NLRP3, ASC, and caspase-1 gradually increased after infection (Figure 3C, Figure 4C and Figure 5C), as did the mRNA of IL-1β (Figure 2A). A previous study suggested that active caspase-1 was an IL-1β converting enzyme, and directly promoted the maturation of IL-1β [18]. Moreover, IL-1β is a common pro-inflammatory cytokine that is deemed to be an index of the severity of inflammation. Thus, we considered that the NLRP3 inflammasome was activated in this model and its expression level was positively correlated with the severity of acute rhinosinusitis at the gene level.

The activation of the NLRP3 inflammasome could be induced by many factors, such as pore-forming toxins and α-hemolysin produced by *S. aureus* [19,20]. Once these agonists enabled the NLRP3 inflammasome, caspase-1 was activated and induced the maturation and release of IL-1β and IL-18, resulting in inflammation. However, the mechanism was considered to be controversial. Three hypotheses on the mechanism of activation of the NLRP3 inflammasome have recently been proposed. The first hypothesis was that the P2X7 ion channel was activated, potassium ions leaked out, resulting in cell membrane perforation, and then the agonists entered the cytosol to activate the NLRP3 inflammasome. The second hypothesis suggested that the lysosome was activated by the agonists and released cathepsin B, which combined with NLRP3 to activate the NLRP3 inflammasome. The third hypothesis proposed that the agonists caused the formation of reactive oxygen species, which activated the NLRP3 inflammasome [17]. In our study, we observed that the NLRP3 inflammasome was activated and a continual increase in the NLRP3 inflammasome after inoculation with bacteria. We presumed that the NLRP3 inflammasome was activated resulting in an inflammatory cascade after inoculation, and then some endogenous NLRP3 agonists, for example, adenosine triphosphate and glucose, were released and activated more NLRP3 inflammasomes [19,21]. In addition, our previous study found that the probability of the formation of biofilm was increased after the mice were inoculated, and thus the pathogenicity of *S. aureus* could be enhanced due to the protection of the biofilm [22].

An interesting phenomenon was that ASC and procaspase-1 had reduced protein and mRNA levels one day after inoculation (Figure 4 and Figure 5) compared with the control group (no bacterial inoculation). Similar results showing a decrease in ASC levels after cytokine induction by *Porphyromonas gingivalis*-infected human THP1 monocytic cells were reported by Taxman *et al.* [23]. We speculated that some substances or some pathways depressed the levels of ASC and procaspase-1. A previous study found that pathogens activated inducible nitric oxide synthase (iNOS) to produce nitric oxide (NO) in the macrophages of mice, which could depress the activation of the NLRP3 inflammasome by preventing ASC pyroptosome formation and inhibiting the activation of caspase-1 [24]. Moreover, it has been reported that *S. aureus* could induce the production of NO by activating the iNOS [25]. We therefore inferred that NO was produced after the activation of iNOS in our experiment, resulting in the prevention of ASC pyroptosome formation and the inhibition of caspase-1 activation. In addition, procaspase-1 could be directly inhibited by superoxide [26]. This type of material could also explain the decrease in procaspase-1 after inoculation compared with controls. The later increases in the expression of ASC and procaspase-1 might be the outcome of the inhibiting effects of these two components could be diminished by decreasing of NO, while decreasing of NO maybe due to increased adenosine triphosphate depleted iNOS [24,27]. The pro-inflammatory effects enhanced by increased adenosine triphosphate and bacterial biofilm formation also could have contributed to the later increases in the expression of ASC and procaspase-1. However, IL-1β mRNA increased at the same time, possibly because not only the NLRP3 inflammasome but also another factor enhanced the expression of IL-1β, such as proteinase-3 and elastase [28]. The exact explanation still needs to be explored in further studies.

## 3. Experimental Section

### 3.1. Animal Models

Ninety-five male C57BL/6 mice (aged 6–8 weeks, 18–20 g body weight) were purchased from the Animal Experimental Center of Wuhan University, Wuhan, China. Upon receipt, the mice were handled under identical husbandry conditions and fed certified commercial feed for 1 week to ensure acclimatization before the first treatment. All mice were randomly divided into five groups, except for five that were used in a preliminary experiment. Eighteen mice were randomly selected as the control group (group A), and were killed with an overdose of anesthetic 1 week after acclimatization. The remaining 72 mice were randomly divided into four groups of 18 mice each, and were treated as described previously [22]. Briefly, the mice were placed under anesthesia by an intraperitoneal injection of a mixture of ketamine (80 mg/kg of body weight) and chlorpromazine (8 mg/kg of body weight), then a glass capillary tube with an inside diameter of 0.9 mm and an outside diameter of 1.2 mm was used to transfer an expansive medical sponge stick into the right nasal cavity, using an insulin syringe with a shortened blunt needle. A 10 μL *S. aureus* suspension (1.2 × 10^9^ CFU/mL) was dropped into the right nasal cavity. These four experimental groups were classified as follows: mice were killed with an overdose of anesthetic 1 day after inoculation (group B), 3 days after inoculation (group C), 7 days after inoculation (group D), and 14 days after inoculation (group E). The animal studies were performed in accordance with the guidelines for the care and use of laboratory animals prepared by the Institution of Laboratory Animals of Huazhong University of Science and Technology. The protocol was approved on 13 June 2013 by the Committee (The Institutional Animal Care and Use Committee of Tong-ji Medical College, Huazhong University of Science and Technology) on the Ethics of Animal Experiments of Huazhong University of Science and Technology (Permit Number: S304).

### 3.2. Bacterial Strain

*S. aureus* strain ATCC 25923 was used in the experiment, and was obtained from the Type Culture Collection in China Center. The strain was stored at −80 °C before use. *S. aureus* was bred on sheep blood agar at 37 °C for 24 h, and then suspended in 1 mL of sterile saline. The suspension was diluted to 1.2 × 10^9^ CFU/mL with an inoculum equivalent to a No. 4 McFarland turbidity standard.

### 3.3. Histological Examination and Immunofluorescence Assay

After the mice were killed, the snouts were obtained and fixed in 4% formaldehyde-phosphate buffered solution. The snouts were decalcified with 10% ethylenediaminetetraacetic acid-sodium, embedded in paraffin, and sliced into 5 μm sections. To determine the inflammation and morphological changes in the nasal cavity, hematoxylin-eosin staining was performed on the sections after deparaffinization and rehydration.

After deparaffinization, rehydration, antigen retrieval and non-specific antigen site blocking, immunofluorescence was performed on the sections overnight at 4 °C with primary polyclonal antibodies (NLRP3, 1:100, Santa Cruz, Dallas, TX, USA; ASC, 1:100, Abclonal, Cambridge, MA, USA; caspase-1, 1:100, Biovision, Milpitas, CA, USA), which were then incubated for 30 min with the secondary antibody Dylight 594 (1:1000, Jackson, West Grove, PA, USA) and mounted in 4',6-diamidino-2-phenylindole dilactate staining. Negative controls were stained similarly after phosphate-buffered solution was used instead of a primary antibody. Images were taken with a laser scanning confocal microscope (Nikon, Tokyo, Japan).

### 3.4. Western Blot

After decapitation, the nasal mucosa was removed from the right nasal cavities of the mice and the total protein of nasal mucosa tissue was extracted using radioimmunoprecipitation assay lysis buffer (Beyotime, Shanghai, China) according to the manufacturer’s instructions, and the protein concentration was determined using a BCA protein assay kit (Beyotime, Shanghai, China).

Protein samples (20 μg for each sample) were separated by sodium dodecyl sulfate-polyacrylamide gel electrophoresis (10%) and subsequently transferred onto polyvinylidenedifluoride membranes (Bio-Rad, Hercules, CA, USA). The membranes were then blocked with 5% non-fat milk at 4 °C overnight, incubated with primary antibodies (NLRP3, 1:200, Santa Cruz, Dallas, TX, USA; ASC, 1:800, Abclonal, Cambridge, MA, USA; caspase-1, 1:1000, Epitomics, Burlingame, CA, USA; caspase-1 (p20), 1:200, Biovision, Milpitas, CA, USA; IL-1β, 1:200, Boster, Wuhan, China; β-actin, 1:2000, Antgene, Wuhan, China) at a working dilution at 4 °C overnight, and subsequently incubated with a solution of horseradish peroxidase-conjugated secondary antibody (1:3000; Antgene, Wuhan, China) for 1 h at room temperature. The membranes were incubated in ECL solution (Pierce Biotech Inc., Rockford, IL, USA), and the gel images were captured using film (Kodak, Rochester, NY, USA), and analyzed using a gel image system (Quantity one) (Bio-Rad, Hercules, CA, USA) to estimate the integral optical density of the protein bands.

### 3.5. Tissue Sample Preparation and Real-Time PCR

After the mice were killed, the nasal mucosa of the right nasal cavities was removed and the total mRNA of the nasal mucosa tissue was extracted using an E.Z.N.A™ Total RNA Kit (OMEGA, Norcross, GA, USA), according to the manufacturer’s instructions. The reverse transcriptase reaction was performed using a PrimeScript™ RT Reagent Kit with a gDNA Eraser (Perfect Real Time) (Takara, Dalian, China). The cDNA was used for real-time PCR analysis using SYBR^®^ Premix Ex Taq™ II (TliRNaseH Plus) (2× Concentration) × 1 (Takara, Dalian, China) according to the manufacturer’s instructions. Primer sequences, PCR cycles and conditions were as follows: *β-actin*: Sense 5'-CTGAGAGGGAAATCGTGCGT-3', antisense 5'-CCACAGGATTCCATACCCAAGA-3'; *Nlrp3*: Sense 5'-TCTTCTCAAGTCTAAGCACCAAC-3', antisense 5'-ACAGCAATCTGATTCCAAAGTC-3'; *Asc*: Sense 5'-CTTAGAGACATGGGCTTACAGG-3', antisense 5'-CTCCAGGTCCATCACCAAGTAG-3'; *Caspase-1*: Sense 5'-ACACGTCTTGCCCTCATTATCT-3', antisense 5'-TTTCACCTCTTTCACCATCTCC-3'; *Il-1β*: Sense 5'-CTTCAGGCAGGCAGTATCACTC-3', antisense 5'-TTGTTGTTCATCTCGGAGCC-3'. These primers were all synthesized by Introgen Co., Ltd. (Shanghai, China). The cycling conditions were polymerase activation for 5 min at 95 °C, 40 cycles of amplification at 95 °C for 20 s, 56 °C for 30 s and 72 °C for 45 s. A cDNA fragment of *β-actin* was amplified as the control. The data were analyzed using the 2^−ΔΔ*C*t^ method.

### 3.6. Statistics

The data were expressed as means ± standard deviations. One-way analysis of variance was used for comparisons of the relative expression levels for the different groups. The software used for statistical analysis was SPSS 15.0 (SPSS Inc., Chicago, IL, USA) for Windows. A *p* value <0.05 was considered to be statistically significant.

## 4. Conclusions

In conclusion, we demonstrated that the expression of the NLRP3 inflammasome was up-regulated over time in the mouse model of acute rhinosinusitis. Moreover, the morphological changes and the changes in IL-1β expression suggested that the degree of inflammation also increased with time, and that the NLRP3 inflammasome might be involved in the development of acute rhinosinusitis. This result may offer a new direction for studying the underlying mechanisms of inflammatory respiratory diseases. In addition, research on the regulation of the inflammasome will probably provide new pharmaceutical targets for acute rhinosinusitis.
